# Synthesis of Novel Cobalt-Containing Polysilazane Nanofibers with Fluorescence by Electrospinning

**DOI:** 10.3390/polym8100350

**Published:** 2016-10-17

**Authors:** Qian Zhang, Dechang Jia, Zhihua Yang, Xiaoming Duan, Qingqing Chen, Yu Zhou

**Affiliations:** Institute for Advanced Ceramics, School of Materials Science and Engineering, Harbin Institute of Technology, Yikuang Street, Nangang District, Harbin 150001, China; cissyzq1011@163.com (Q.Z.); xmduan@hit.edu.cn (X.D.); 13B909014@hit.edu.cn (Q.C.); zhouyu@hit.edu.cn (Y.Z.)

**Keywords:** polycobaltsilazane, electrospinning, nanofibers, luminescence

## Abstract

Emission in the nanostructured materials is important in micro/nanoelectronic devices. We report here a strategy for the processing of micron and submicron fibers from a cobalt-containing hyperbranched polysilazane by electrospinning. The electrospun nanofibers have uniform average diameters of ~600 nm and lengths of ~10 μm. The photophysical properties of polycobaltsilazane (PCSN) are studied using UV-VIS and photoluminescence spectroscopies. PCSN fibers display a series of emission peaks between 490 and 615 nm. The Co(II) doping into polysilazane leads to the emission from 465 to 415 nm. The emission wavelength shift of Co(III)-containing polysilazane is specific under 340 and 470 nm excitation wavelengths, respectively, while it is not observed with metal-free polysilazane. Thermogravimetric analysis-Differentical thermal analysis (TGA-DTA) profiles also show good thermostability of the PCSN fibers at 800 °C under Ar atmosphere. The use of PCSN offers both enhanced ceramic yields against ~5 wt % starting material and the fluorescence intensity of polymeric fibers.

## 1. Introduction

Currently, selected silicon-based hyperbranched polymers have been shown to offer semiconducting behavior that combine the optical and electronic properties of organic semiconductors with the processing advantages and mechanical properties of polymers [1,2,3,4,5]. These materials have been employed in chemical sensor applications based on fluorescence sensitivity towards various anions [6,7], cations [8], as well as amine complexes [9]. Although the operational mechanisms of inorganic photodetectors operate vary, most of polymer-based photodetectors perform by converting absorbed photons into free charge carriers [3]. Furthermore, many hyperbranched polymers are fluorescence materials with a Stoke shift which separates emissions sufficiently far from the absorption edge to minimize self-absorption. Therefore, semiconducting luminescent polymers offer promise as an interesting material for fluorescence [1].

Polysilazane polymers to silicon nitride and nitride carbide fibers offer potential as ceramic precursors but are also possible as semiconductor precursors with optical and electronic properties [10]. Polysilazane affords strong absorption over a wide range of the UV region as well as efficient fluorescence, which are attributed to the delocalized electron along the Si–N skeleton [11]. Moreover, the introduction of late transition metals to the polymer chain can be used to modify precursors to introduce cross-linking and form organometallic dendrimers, thus improving the shape retention for 2D and 3D materials [12,13].

The first study on the reactions between polysilazanes and metal complexes were reported by Seyferth and coworkers [14]. The presence of Si–H and Si–vinyl in the polysilazane provided an opportunity to add polynuclear transition metal species to the polymer backbone. It has been also suggested that the incorporation of heteroatom and amido functional groups into the skeleton of polysilazane could improve the polymeric hyperbranched degree and form a class of hyperbranched metallopolymers resulting in the increased fluorescence [15]. Due to an efficient cobalt-catalyzed transformation of organic molecules and mechanical properties after pyrolysis, previous studies mainly focused on iron-containing hyperbranched metallopolymers and the amido application of such materials is achieved through controlling the conformation changes under different conditions [11,16,17,18,19]. In the context of metallopolymers little work dealing with cobalt(II)-containing systems has been published, while in the case of the modification by coordination compounds, the utility of amino complexes is quite effective.

The exploitation of suitable deposition methods is crucial to exploit the potentiality of metallopolymers. Electrostatic or electro-spinning can be used to reduce polymer fiber diameters [20,21], and has been used to process fibers for various applications in fields such as energy, healthcare, and environmental engineering [22,23,24]. In fact, electrospun fibers also have become a promising versatile technology for the separation of emulsified oil/water mixtures because of the high specific surface areas, interconnected open pore structure, and the potential to incorporate active chemistry on nanosurfaces [25,26]. Electrospun fibers possessing π-conjugated backbones and π-conjugated semiconductor polymers are expected to offer potential utility in developing photoluminescent materials and functional fabrics for smart textiles, opto- and nanoelectronic, as well as piezo- and thermoelectric generators [24,27,28,29]. In this study, we discuss the synthesis of electrospun nanofibers composed of cobalt-containing polysilazane with a large diameter and a continuous-fiber electrostatic spinning method. Furthermore, the fibers of hyperbranched polymer are fabricated directly from only a polycobaltsilazane (PCSN) solution without the use of additional polymers or stabilizers. Our experimental results prove that polycobaltsilazanes are a processable class of high molecular weight metallopolymers which are readily and facilely available by a self-assembly polymerization route. These materials result in polymer shaped, functional electrospun nanofibers on the macro-, micro- or nanoscale [30,31].

## 2. Materials and Methods

### 2.1. Materials

The poly[(methylvinylsilazane)-*co*-(methylhydrosilazane)] (PSN) was purchased from Gelest Inc. Commercial polysilazane (The Institute of Chemistry, Beijing, China). *N*,*N’*-dimethylformamide (DMF), toluene, CoCl_2_·4H_2_O, and ethylenediamine were purchase from Aldrich (Sigma-Aldrich, Yongin-si, Korea). The PSN was used as-received without any further purification, tris(ethylenediamine-k^2^N)cobalt(III) chloride ([Co(en)_3_]·Cl_3_) was synthesized by CoCl_2_, and ethylenediamine was used as described previously [32,33]. Anhydrous tetrahydrofuran (THF) was freshly distilled under reflux using sodium/benzophenone in Ar, while calcium hydride was used as a drying agent to remove water in DMF and toluene.

### 2.2. Characterization

Field-emission scanning electron microscopy (SEM) and energy dispersive spectroscopy (EDS) analyses were taken on a Quanta 200FEG instrument (FEI, Hillsboro, OR, USA). The specimens were treated by spraying a thin layer (1–2 nm) of gold (EMITECH K550X, Kent, UK) before the SEM test. This instrument is also equipped with a focused-ion beam (FIB) to perform the resultant analysis in detail. The samples for transmission electron microscopy (TEM) were prepared though electrospinning of fibers using polymer as the liquid source fellowed by in situ deposition of the sample on carbon-coated copper. TEM (FEI, Tecnai G2F30, FEI, Hillsboro, OR, USA) was operated at 200 kV, coupled with electron diffraction analysis. SEM and TEM were used to study the microstructure and chemical nature of the nanofibers.

X-ray photoelectron spectroscopy (XPS) measurements were conducted on an AlK*α* (*hv* = 1486.6 eV) spectrometer (Kratos, ULTRA AXIS DLD, Kratos, Tokyo, Japan), and the analyzed area was 800 mm in diameter for analyzing the electronic structure of specimens. Binding energies were calibrated to reference the C 1s line at 284.6 eV, and the curve fitting of the XPS spectra was performed using the least-squares method. The Fourier transformed infrared (FT-IR) spectra were collected in transmission on a Perkin-Elmer Spectrum 100 spectrophotometer (Perkin-Elmer, Waltham, MA, USA). The FT-IR spectrum was recorded between 500 and 4000 cm^−1^, and the samples were prepared as a KBr pellet. The molecular weights of the polymers were determined by gel permeation chromatography (GPC) with an Agilent 1260 instrument and miniDAWN TREOS using THF as a calibrated against a polystyrene (PS) standard.

Simultaneous thermogravimetric analysis and differential scanning calorimetry coupled with mass spectrometry was performed using a simultaneous thermoanalyzer (STA 449 C) coupled with a quadrupole mass spectrometer (QMS, 403 C Aëolos (Netzsch Group, Selb, Germany) at a temperature range of 40–1500 °C, with a heating rate of 10 °C·min^−1^ under an argon atmosphere (gas flow: 50 mL·min^−1^).

The fluorescence morphology of the nanofibers was studied using a polarized light microscope (Olympus BX-51, Olympus, Tokyo, Japan). For the measurement of the visible photoluminescence (PL) emission spectra, the PL spectra were recorded on a FLS 920 combined steady state fluorescence and luminescence spectrometer (Edinburgh Instruments, Livingston, UK) using a xenon lamp as the excitation source at ambient temperature. The sample was placed in a 10 mm four-sided polished quartz cuvette to measure the real emission intensities.

### 2.3. Synthesis of Co/Polysilazane

**Figure 1:**
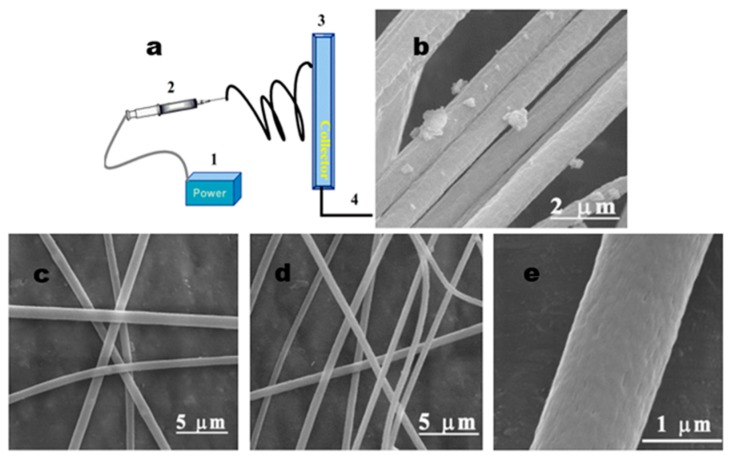
(**a**) Schematic representing of the electrospinning setup (1: power source, 2: the syringe with polymer solution and the needle, 3: collection target, and 4: wires attached to the power source); (**b**–**e**) SEM images of electrospun fibers of 20 wt % PCSN solution.

We have recently reported the synthesis of polyborosilazane nanocomposites at room temperature during the course of reaction the PSN, BH_3_, BCl_3_, and [Co(en)_3_]·Cl_3_. The synthesis of the polymer was carried out using the standard Schlenk technique under Ar. The reaction between the Co(II) complex and PSN causes the release of some polysilazane fragments in the form of ammonia (see Figure 1) [34]. The polycobaltsilazane used here is the result of the reaction between 15 wt % Co(II) complex and the polysilazane in DMF. The solution was stirred at 120 °C for 12 h under an Ar atmosphere. After removing DMF under vacuum, a cobalt-containing polymeric precursor was obtained. The elemental composition of the thermolysis product of the recovered polycobaltsilazane at 800 °C (the details of the thermolysis analysis is mentioned later) indicates Si, C, N, and Co content of 33.95, 30.32, 28.55, and 7.18 wt %, respectively, corresponding to an empirical formula of C_18_H_53_CoN_15_Si_9_.

The approach involved a two-stage process in which the formation of the well-ordered microphase-separated structure of block copolymer was first achieved and then, using a selective solvent, the self-assembled domain was isolated. The main requirement here was that during solvent treatment, the self-assembled domains should not undergo any distortion/deformation. Hence, the used solvent should be strongly selective for the matrix-form blingock.

For the electrostatic spinning experiments, the polymer was dissovled in DMF and stored in a plastic syringe with a 0.8 mm diameter blunt needle, and the amount of solvent was adjusted to achieve a suitable viscosity of the spinning solutions. The most promising results were obtained using a 30 wt % solution of polycobaltsilazane in DMF. Electrospinning experiments were carried out in an Ar atmosphere, with a constant gas flow to expel the evaporating solvent. The effects of moisture and oxygen should be rigidly controlled in the process of electrospinning, so the spinning equipment and collector were placed in a plexiglass cover.

## 3. Results and Discussion

### 3.1. Synthesis of the Polymer Precursors

**Scheme 1:**
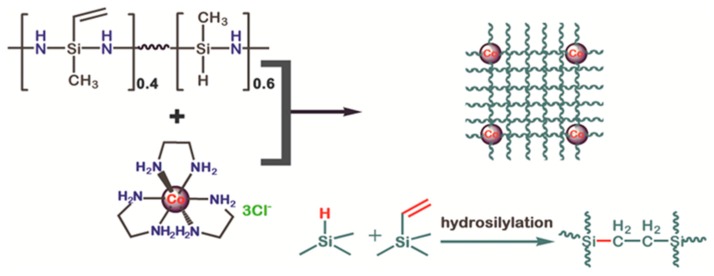
The approach involved in the synthesis of polycobaltsilazane: polysilazane reacted with the Co(III)-complex in DMF to form a conjugated polymer, polycobaltsilazane.

Novel cobalt-containing polymeric precursors for the polymer fibers were successfully prepared from a polysilazane (PSN) containing Si–H bonds and vinyl groups [34,35]. Spectroscopy was used to characterize the polymer, which has verified the structure and chemical connectivities as schematically shown in Scheme 1. As is already known, PSN can form a cross-linked network via hydrosilylation or free-radical polymerization below 200 °C [15]. The title material, polycobaltsilazane, was prepared via a reaction of the [Co(en)_3_]·Cl_3_ complex with the polysilazane with a high density of terminal hydrogen groups in the absence of a catalyst, with a *M*_w_ of 4000 g·mol^−1^. In this reaction we presume that the complex is first reduced to Co metal clusters that then promote cross-linking via hydrosilylation.

### 3.2. Polymer Nanofibers

The resulting polycobaltsilazane is still soluble (DMF) and allowed electrospinning of continuous microfibers using the setup as described in Figure 1 (the electrospining system shown with an aluminum foil as the collector). Figure 1a shows a schematic diagram of the electrospining process for the continuous fiber. Since the precursor concentration is known to strongly influence electrospinnability of the compounds [24,36], various polymer concentrations were tested to determine an optimized viscoelastic behavior of the solution, while the morphology and fiber diameter of electrospun fibers were also influenced by voltage, distance between the needle and target, as well as the solvent. In all of the experiments, by using a high voltage supply, the applied voltage to the metal needle tip is 15 kV. In addition, the polycobaltsilazane exhibits poor solubility in a number of solvents except for DMF. Therefore, electrospinning was carried out with a voltage of 15 kV and a flow rate of 55 μm·min^−1^. A piece of aluminum foil is used to collect the fibers, and the distance to the nozzle was 10 cm.

**Figure 2:**
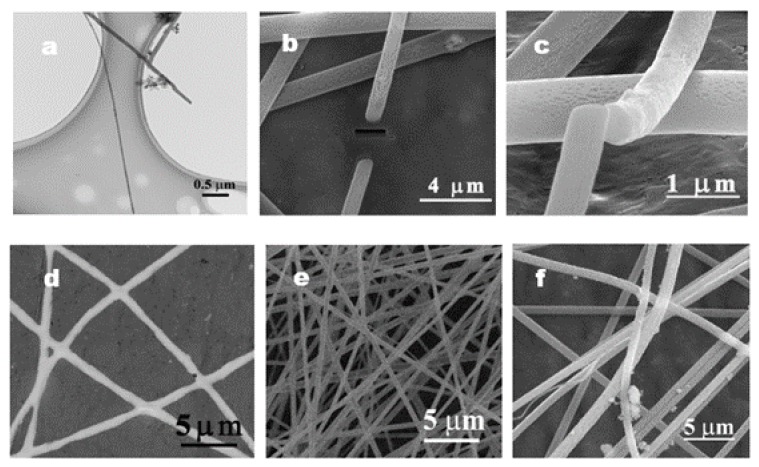
(**a**) TEM image of the polymer fibers; (**b**,**c**) FIB images of polycobaltsilazane nanofibers; (**c**) the porous nature and cross-sectional view of the nanofiber; (**d**) SEM image of the nanofibers at PCSN/DMF ratios of 12 wt %; (**e**) SEM image of the nanofibers at PCSN/DMF ratios of 20 wt %; and (**f**) SEM image of the nanofibers at PCSN/DMF ratios of 25 wt %.

The SEM images of the electrospun fibers indicate an average diameter of <1 micron. Figure 1b shows the morphologies of polycobaltsilazane electrospun fibers. After the solvent evaporated, fibers with micron scale diameters were found. Figure 1c displays collected fibers which are spread out and branched off in many spreading directions from the tip edge of the flat collector. The 12 wt % polymer solutions are not suitable for spinning and resulted in electrospraying, powder, or fibers with a beaded morphology (Figure 2d,f). From Figure 1d, it can be observed that the uniform fibers were formed at a higher concentration of 20 wt %.

To the best of our knowledge, we reported the first report of a sinlge-step spinning of a polysilazane derivative. Figure 2 showed that fiber diameters range between 600 and 900 nm. The best electrospun fibers showed no necking or nodes at a higher concentration compared with that at the lower concentration. Therefore, the polymer/DMF ratio of 20 wt % was selected to obtain slimmer fibers, compared with lower concentrations (12 wt %) in the same sample.

Figure 2 presents TEM images and the SEM (FIB) analysis of electrospun Co-containing polymeric fibers, the electrospun fibers which are prepared by the polymeric solution concentration of 12 wt % (see Figures S1 and S2). It is evident in the SEM characterization that the diameter of the electrospun fibers is discontinuous, exhibiting a low throughput, as well as presenting random nodules due to the weak adhesive force.

When the concentration was increased up to 20 wt %, the diameter of fibers had no distinct changes. The FIB analysis showed well-formed homogeneously-structured fibers with an average diameter of 600 nm, whereas differences in the fiber morphology appeared; for instance, the fibers‘ surface became smoother and the thickness of fibers was more homogeneous than that of low concentrations (Figure 2a–c).

In the PCSN 25 wt %/DMF solution there were more obvious differences in fiber morphology, as shown in Figure 2d. The fibers were flat and conglutinated together as a dense sheetlike network. Furthermore, the fibers were toughened enough and of a smooth texture before they were collected. The observed interconnectivity was expected to stabilize the three-dimensional structure, which was important for a high conjugation desired for functional application [29,37].

### 3.3. Structure Analysis

**Figure 3:**
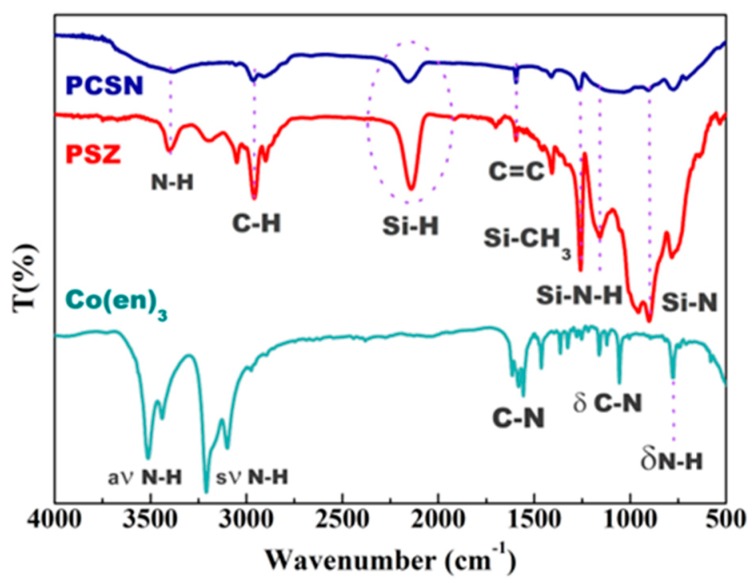
FT-IR spectra of polysilazane (PSN) and with 20 wt % Co(en)_3_-containing polysilazane (PCSN). FTIR spectra of [Co(en)_3_]·Cl_3-_modified polysilazane (blue line), polysilazane (red line), and Co(en)_3_ (cyan line). The strong absorptions from 1178 to 1080 cm^−1^ correspond to Si–CH_2_.

The FT-IR, SEM, and XRD characterization of resultant cobalt-polysilazane compounds exhibits great influence on the starting polysilazane via the reaction with the [Co(en)_3_]·Cl_3_ complex. Basically, there are three main effects involved. First, the reaction between the –NH groups of the ethylenediamine coordinated by the Co(III) complex with the silicon centers of the polysilazane. Second, the hydrosilylation reaction takes place between Si–H and Si–Vi. Third, the high density of hydrogen bonds on the termini makes it possible to the incorporate Co(III) complex into the cross-linked networks of PS by hydrosilylation in the absence of a catalyst (Co(III) ions). As analyzed from the FTIR spectra, the reaction that occurred at both N–H and Si–H functional groups takes place only at the N–H groups [38]. FT-IR spectra of the pure polysilazane and after the reaction with Co[(en)_3_]·Cl_3_ are shown in Figure 3. The typical absorption bands related to N–H (3360 cm^−1^), Si–N–H (1160 cm^−1^), Si–N (950 cm^−1^), C=C (1609 cm^−1^), C–H (2960 cm^−1^), and Si–H (2124 cm^−1^) are shown. The evidence that the formation of a polycarbosilane network is responsible for the strong absorption bands located at 1255 cm^−1^ belong to Si–CH_3_ groups. It may also arise from residual comparisons with the FT-IR spectra of PSN, and the absorption bands related to N–H (3360 cm^−1^) and Si–H (2124 cm^−1^) groups, indicating that the reaction occurs at the N–H and Si–H substituents of PSN; whereas the reaction with the cobalt amido complex did not affect the vinyl groups. A similar reaction at both N–H and Si–H groups of the PSN was also shown in the reaction of hafnium amido complex with HTT 1800 [35].

**Figure 4:**
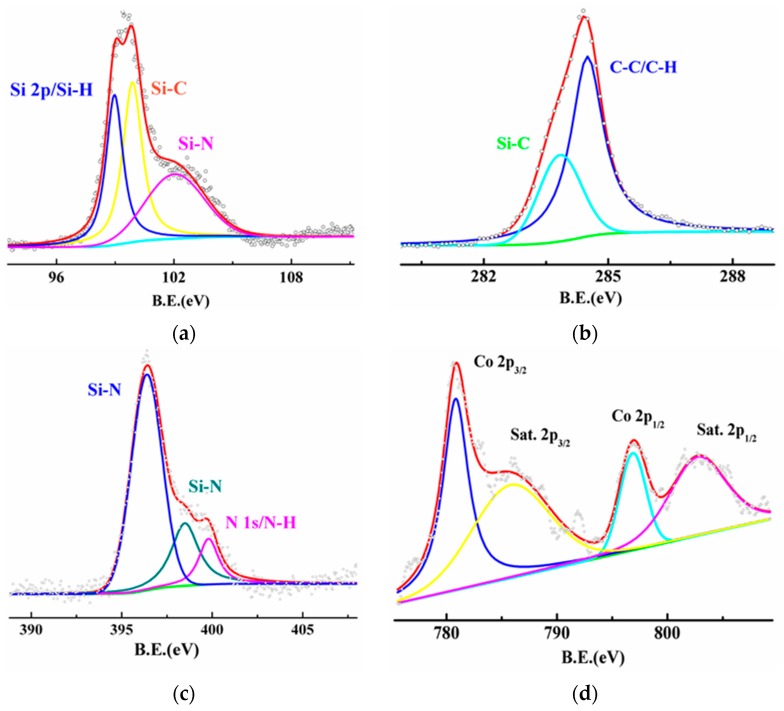
The fitting curves of XPS analysis of polycobaltsilazane. (**a**) The spectra of Si 2p reveals the Si–C bonding (yellow line), Si–H bonding (blue line) and Si–N bonding (pink line); (**b**) the spectra of C 1s reveals the C–C bonding (blue line) and C–Si bonding (Cyan line); (**c**) the spectra of N 1s reveals the Si–N bonding (Dark Cyan and blue line) and the N–H bonding (pink line); (**d**) the spectra of Co 2p reveals the characteristic of Co(II) ions (blue and Cyan line) and Co 2p3/2 (yellow line) and 2p1/2 (pink line) satellite structures. The spectrum curves (open circles) are deconvoluted by Gaussian fitting (red solid curves).

To further determine the chemical composition, X-ray photoelectron spectroscopy (XPS) of polycobaltsilazane was performed. Figure 4 showed three typical peaks corresponding to the binding energy of C 1s, N 1s, Si 2p, and Co 2p. The high-resolution XPS spectrum of C 1s in Figure 4a revealed that three distinct peaks could be fitted at 283.4, 284.5, and 286.3 eV, which were attributed to Si–C, C–H, and C–N bonds, respectively. The C=C bonds (sp^2^ type carbon) at 284.5 eV was the major peak [33,39]. The other peaks are due to C–N species at 286.3 eV and Si–C at 283.4. The silicon peaks at 100.4 eV [33,40] and 102.0 eV [41] corresponding to Si–C and Si–N bonds, respectively. Meanwhile, the N 1s band on deconvolution showed peaks at 396.8 eV and 397.8 eV attributable to Si–N bonds [40].

Figure 4d exhibits the Co(II) 2p binding energies at 780.8 and 797.2 eV, characteristic of Co(II) ions, and the intense Co 2p_3/2_ and 2p_1/2_ satellite structures centered at 786.0 and 802.8 eV, respectively [42]. In addition, Table 1 indicates that the N content of the Co complex was slightly higher than that of PSN. This is likely due to partial incorporation of the amine ligands into the polymer as supported by the commensurate increase in carbon content. The quantitative analysis illustrates that the C, N, Si, and Co atom contents were 33.35, 27.75, 28.67, and 7.36, respectively. Results clearly showed that the percentage of silicon in the silicon-functionalized specimen (33.35 wt %) was significantly lower than that of the without-cobalt specimen (43.61 wt %). Therefore, the cobalt complex was successfully attached to the polysilazanes.

### 3.4. Thermal Stability

**Figure 5:**
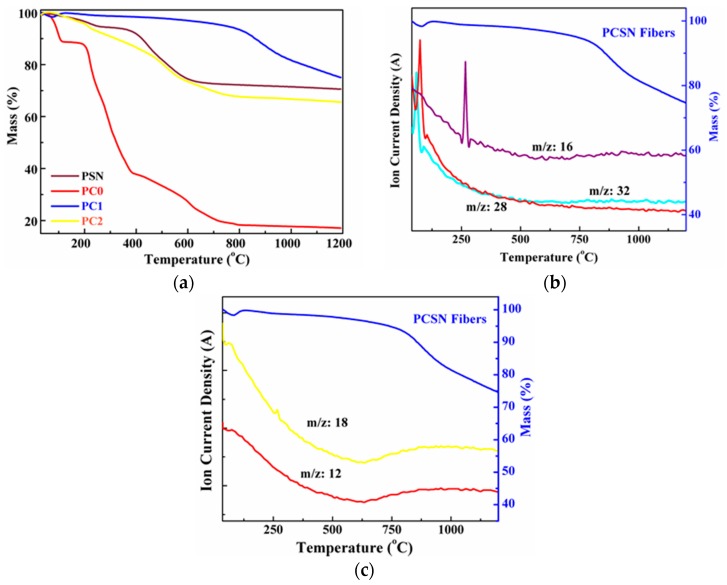
Polymeric fiber thermograms obtained from simultaneous thermal analysis of TGA-DSC under Ar at a scanning rate of 10 K·min^−1^. (**a**) Thermogravimetric analysis; and (**b**,**c**) mass spectrometry quadrupole mass spectrometer (QMS) ion current curves of cross-linked polysilazane modified with 20 wt % Co(en)_3_ showing the release of (**b**) hydrogen and methane; (**c**) ethane, and ethylamino fragments during the thermal degradation process.

TGA-DSC coupled with QMS was used to investigate the cross-linking and pyrolysis behavior of these polymers. Figure 5 indicated the TGA and DSC studies (Ar) of PSN, Co complex (PC0), and polycobaltsilazane (PC1) and (PC2) with 12 and 20 wt % Co(III) complex. From the TGA curves, it can be observed that PC0 underwent a rapid thermolytic degradation in the temperature range of 200 to 550 °C, while there is no further mass loss observed by QMS analysis after that. The weight loss ratio under these condition is 80 wt %, which has the most mass loss among these specimens.

However, with the mass loss of the compound of the Co(III) complex and PSN appear interesting results that offer quite a different behavior to the PSN (the mass loss between 12 and 20 wt % increases with Co content). A possible reason could be the enhanced cross-linking promoted by the Co(III) complex [35,43]. In this case, the dendrimers are the cross-linked points with the cross-linked interface between PSN and Co[(en)_3_]·Cl_3_, as described in Scheme 1. Meanwhile, the shift of the DSC endothermic reaction to a lower temperature demonstrates this as well, as a 5 wt % decrease was found in the heat enthalpy for P1 and P2 (Figure 5). However, because of an increased thermolytic degradation of Co(III)-complex, the increase in the mass loss suggests that the pyrolysis of Co[(en)_3_]·Cl_3_ is altered on account of the extensive cross-linking via the hydrosilylation or dehydrocoupling reaction. This can be further identified by QMS spectrometry, as follows.

Figure 5 indicates the difference among the polymers of PSN, Co[(en)_3_]·Cl_3_, and polycobaltsilazane through the result of TGA coupled with QMS. Above 800 °C, no mass loss is seen as the decomposition of polycobaltsilazane is completed up to this temperature. The thermolysis of PC1 is mainly accompanied by the evolution of H_2_ (*m*/*z* = 2), CH_4_ (*m*/*z* = 16), NH_3_ (*m*/*z* = 17), as well as oligomer fragments (*m*/*z* = 28) and (*m*/*z* = 32). The cross-linking of the Co-containing polymer relies mainly on the hydrosilylation and vinyl polymerization processes, as reported also for other Si–H and vinyl-terminated substituted polysilazane-based polymers [35,44,45]. These processes occur without the mass loss and it can be suggested that the polycobaltsilazane is more stable as compared to the Co-free polysilazane. The NH_3_ evolution is due to the transamination reaction causing a weight loss of ~8 wt %.

In addition, transamination processes between ≡Si–N and Co–N/Si–N= groups likely occur with the release of amine fragments, as indicated by mass spectrometry. The thermolysis of the PCSN differs significantly from that of the PSN with respect to both the nature and kinetics of the evolved gaseous species, which is consistent with the claimed structural modification of PCSN. In the decomposition process above 400 °C (Figure 5c), the mass losses arise from the evolution of hydrogen and methane, as well as ethane and ethylamino fragments. The hydrogen release is ascribed to a dehydrocoupling reaction occurring between Si–H and N–H groups, which leads to the formation of a ≡Si–N= linkage. The release of methane and ethane occurs due to the decomposition of the organic substituents of the polycobaltsilazane. Nevertheless, the formation of the diethylamino fragments arises to a great extent by a process of transamination between ≡Si–N= and Co–N= and the decomposition of –NEt_2_ groups in this polymer.

### 3.5. Fluorescence

**Figure 6:**
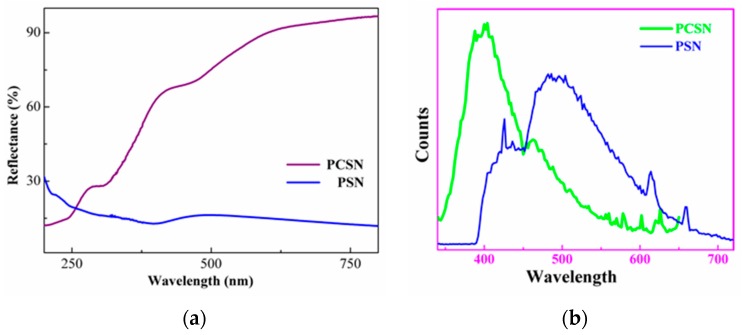
UV-Visible absorption spectra (**a**) and fluorescence (**b**) spectra of PCSN powder at room temperature; and (**b**) normalized photoluminescence excitation and emission spectra of polycobaltsilazane powder. The fluorescence studies were conducted at excitation wavelengths of 340 nm; the maximum emission wavelengths were observed at 390, 415, 485, 580, and 630 nm.

In the present study, the PL emission of the resultant Co-containing polymer is observed under UV-VIS wavelengths at room temperature. The UV-VIS absorbance peaks at 260 and 390 nm are observed to grow in intensity, which are due to polysilane-based transitions, as shown in Figure 6a. Meanwhile, the band at 465 nm has also been observed, which is assigned to the ^4^T_1g_(F) → ^4^T_1g_(P) transition in polysilazane high-spin cobalt(II) complexes [42,46]. The broad band at 465 nm reveals strong intensiyt; it is considered that the cobalt remains predominantly a cobalt(II) ion after cross-linking with PSN. In addition, there is a marked redshift band of absorption (40 nm) at around 260 nm, compared to PSN, based on a change of the geometric structure by the C=N groups and Co atoms. The UV-VIS results are in good agreement with the results from XPS.

The emission and excitation spectra are recorded for the maximum intensity excitation and emission peaks, which are shown in Figure 6b,c, respectively. A significant feature of the PL spectra is the appearance of a new band at 490–510 nm instead of the emission bands associated with polyvinysilane units of the polysilazane [47] due to the wide band gap of polysilazane being ~2.5 eV, which is transparent for green to red light [48]. The photosensitivity of the functionalized polyvinylsilazane was comparatively evaluated by measuring the UV-VIS absorbance spectra [49]. The new emission is found to be independent of the excitation wavelengths in the range of 520–540 nm, thereby suggesting that the obtained emission is a real luminescence from the relaxed state due to the scattering effect [50]. This emissive behavior of the polymer is corroborated by the observed green luminescence under UV-VIS wavelengths (Figure 6).

**Figure 7:**
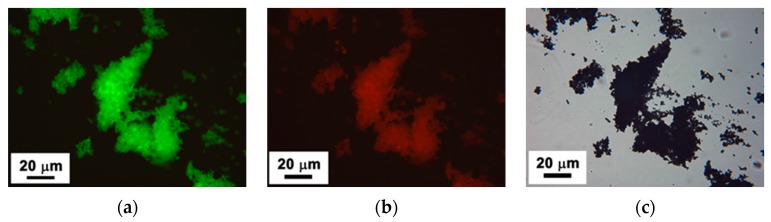
The fluorescence micrographs of some polymer powder on commercially available filter paper under UV light (**a**) and (**b**), as well as daylight (**c**).

Figure 7 shows the confocal laser scanning microscope images of the PCSN powder under UV light, featuring bright light emission. Nevertheless, these photos not only exhibit significant green fluorescence in accordance with Figure 7a, but also exhibit red fluorescence under different UV-VIS wavelengths in Figure 7b, and both of them are quite different from that under daylight in Figure 7c. A plausible explanation of the observed emission maximum at 620–660 nm is provided by considering the nature of the polysilazane bearing different side chain substituents at a 470 nm excitation wavelength. The increase of polymerization degree of the polymer backbone is attributed to the redshift of emission [51]. Furthermore, the excitation wavelength can influence the process of energy transfer in the core-complex of silicon-based polymer. The red emission peaks at 620–750 nm and associated fluorescence characteristics of the polymers are due to cobalt(II) ions. It was demonstrated that the interesting photochromic performance originates from the molecular structure of the polysilazane, which can be naturally considered as an intrinsic defect. The results obtained from the photoluminescence spectra, and the fluorescent nature of the same sample as a great light emitter, provides a strong basis to suggest the formation of polycobaltsilazane.

The above results present that the branched organic silicon-based polymers are versatile carriers for the generation and stabilization of metal complexes as fluorophores [50]. To address the origin of the fluorescence obtained for the cross-linked PCSN, it is necessary to suggest the structural changes and rearrangements associated with the processing temperature. During the curing process, the polymer structure transforms into a 3D network structure. Thus, the formation of defects (e.g., radicals) can occur with the increase in the cross-linking degree. Meanwhile, such materials display the potential of new-generation decorative materials and fascinating stage effects.

## 4. Conclusions

To summarize, we have demonstrated that branched polysilazane undergoes a facile reaction with a Co(II) complex to afford cobalt-containing polymeric fibers which are fluorescent on account of the formation of conjugated polycobaltsilazane. The polycobaltsilazane is formed in the substituting reaction among a polysilazane with vinyl-terminated and Si–H functional groups and a cobalt amino complex via a catalyst-free polymerization reaction. As a result, these newly-constructed polycobaltsilazane have comparable stabilities with undoped polysilazanes, as seen in TGA-DTA analysis, and may have significant implication in synthesis of hybrid noble ceramic nanofibers. Additionally, the PCSN fibers exhibit interesting fluorescence that can be tuned by controlling the excitation wavelengths in the visible range at low temperatures. The fibers show a greenish fluorescent emission peak around 490–510 nm under a 340 nm excitation wavelength, while a strong reddish fluorescence emission was obtained under a 470 nm excitation wavelength. There is a promising application in the study of stimulated emission of conjugated polymeric semiconductor materials.

## Figures and Tables

**Table 1:** The elemental composition of PSN and Co-containing polymer, including an estimation of the carbon content and the sum formula.

Sample	Si (wt %)	N (wt %)	C (wt %)	O (wt %)	Co (wt %)	Sum formula
PSN	43.61	25.74	27.55	3.10	0	Si_1.56_N_1.84_C_2.29_O_0.19_
Polycobaltsilazane	33.35	27.75	28.67	2.87	7.36	Si_1.19_N_1.98_C_2.39_Co_0.125_O_0.17_

## Supplementary Materials

The following are available online at www.mdpi.com/2073-4360/8/10/350/s1, Figure S1: SEM images of electrospun fibers of 12 wt % PCSN solution. It seems that 12 wt % polymer solutions are not suitable for spinning and resulting in electrospraying, powder or fibers with beads morphology, Figure S2: SEM images of electrospun fibers of 12 wt % PCSN solution. Figures S1 and S2 are the same concentration of PSCN, but with different resolution images.
