# Supplementary material for: Synthesis of Novel Cobalt-Containing Polysilazane Nanofibers with Fluorescence by Electrospinning
Source: Polymers (Basel). 2016 Oct 17;8(10):350. doi: 10.3390/polym8100350 (PMC6432467; doi:10.3390/polym8100350)
Supplement: Supplementary file 1 [file polymers-08-00350-s001.pdf]

Supplementary Materials: Synthesis of Novel Cobalt-Containing Polysilazane Nanofibers with Fluorescence by Electrospinning

Qian Zhang, Dechang Jia, Zhihua Yang, Xiaoming Duan, Qingqing Chen and Yu Zhou

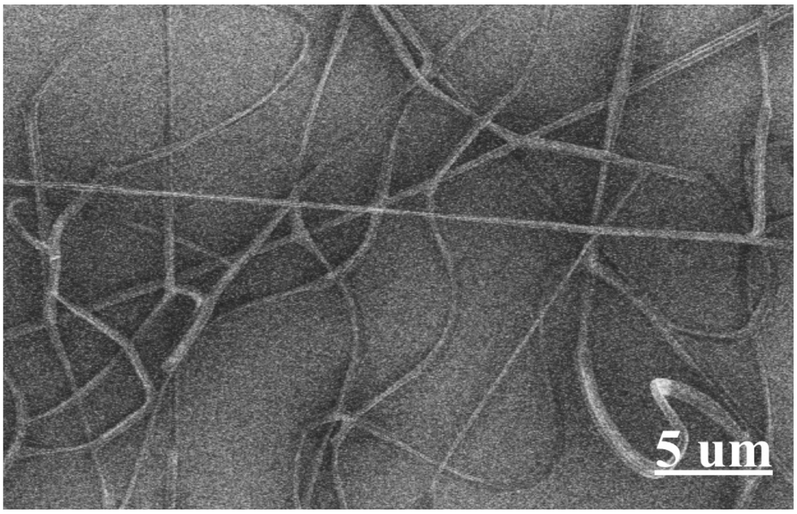

Figure S1. SEM images of electrospun fibers of 12 wt % PCSN solution. It seems that 12 wt % polymer solutions are not suitable for spinning and resulting in electrospraying, powder or fibers with beads morphology.

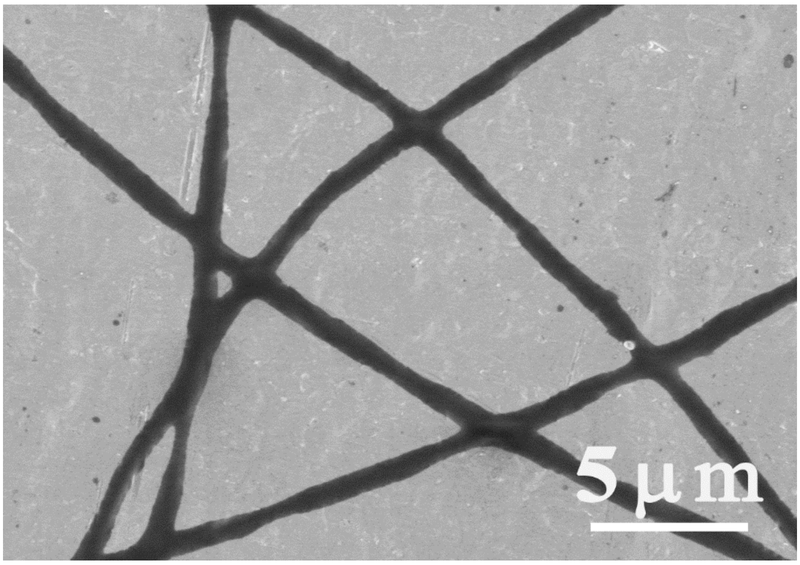

Figure S2. SEM images of electrospun fibers of 12 wt % PCSN solution. Figures S1 and S2 are the same concentration of PSCN, but with different resolution images.
